# Conflict, trust, and power in international joint ventures: a governance perspective

**DOI:** 10.3389/fpsyg.2026.1863432

**Published:** 2026-06-23

**Authors:** Monaf Almahmoud, Xiaokang Zhao, Fawad Ullah, Nyo Me Hlaing

**Affiliations:** Glorious Sun School of Business and Management, Donghua University, Shanghai, China

**Keywords:** conflict management, international joint ventures, joint venture performance and stability, power dynamics, trust

## Abstract

Conflict is an inherent yet controversial aspect in international joint ventures, having two facets: being disadvantageous for collaborations as well as a fuel for learning and adaptation, making it a research interest to assess its performance and stability for researchers and business organizations. This research extends its understanding of conflict as a consequence of the discrepancy between organizational work and the goal set by the owner of the organization. Then, it explores whether conflict affects directly or indirectly, depending on the thing managed or governed. Herein, we test the direct and mediated relationships among conflict, conflict potential, trust, power dynamics, performance, and stability, using survey data from 199 professionals involved in international joint ventures in China and applying Partial Least Squares Structural Equation Modeling. The results suggest that conflict does not completely increase performance but substantially increases trust and power recalibration. Trust as well as power positively induce performance and stability, with power showing a principally strong effect on stability. Conflict potential strongly forecasts trust and exerts indirect effects on consequences through interactive pathways. Hence, conflict can be considered as a governance trigger rather than a direct performance driver. By incorporating the foundation on two well-established theories, i.e., Institutional Theory and Agency Theory, this study proceeds international business research by determining that the impact of conflict in international joint ventures is provisional upon effective relational and structural governance responses. The results in this research present theoretical clarification and practical guidance for managing cross-border partnerships in IJVs operating in China.

## 1 Introduction

International joint ventures (IJVs) occupy a contradictory position in global business strategies (Bhateja and Jayantha, 2026; Rocha et al., 2022), as they allow firms to tap local knowledge, spread out risk, overcome institutional barriers, and enter challenging markets (Liu et al., 2025; Roh et al., 2026). Nonetheless, they are also categorized by high instability rates, changes in ownership arrangements, and premature termination (Gehrisch and Süß, 2023; Wang et al., 2026). At the center of this contradiction lies a perpetual relational tension: collaboration requires mutual reliance, but shared ownership at the same time produces divergent goals, control disagreements, and irregular access to information (Gaba and Joseph, 2023; Lewis et al., 2010; Manca, 2022).

As a result, conflict is structural in IJVs—not an abnormality. Early IJV research has demonstrated conflict as a defining feature, as a key characteristic, of international partnerships (Fey and Beamish, 2001; Habib, 1987). Succeeding scholars demonstrated that differences in knowledge and bargaining strength shifts often undermine ventures over time (Inkpen and Beamish, 1997). At the same time, relationship-focused studies showed that trust can moderate opportunistic behavior and advance cross-border partnership alliances (Arora et al., 2024; Aulakh et al., 1996; Boersma et al., 2003; Hwang and Kim, 2018).

The academic landscape remains theoretically fragmented regarding a fundamental issue: Is conflict favorable or unfavorable to IJV performance and stability? Some studies indicate that constructive conflict can foster learning and adaptation (Ali et al., 2021; Wong et al., 2018), while other findings argue that uncertain and unsolved conflicts erode trust and negatively affect performance (Khosravi et al., 2020). What remains unclear and underdeveloped is a solid theoretical framework of when and why conflict yields positive or negative outcomes.

In this study, trust is the belief that a partner will act reliably, competently, and with goodwill, evolving through promises, competence, and mutual care (Boersma et al., 2003). While trust plays a foundational role in mitigating conflict, power dynamics also shape conflict outcomes in IJVs (Jin and Wang, 2021; Ma and Mihalache, 2026; De Mattos and Salciuviene, 2025; Talay and Akdeniz, 2025). Power is derived from control over resources and decision-making authority, with partners holding power through contractual rights and residual claims that determine control over decisions and resources (Jensen et al., 1976; Reuer et al., 2024; Stoelhorst and Vishwanathan, 2024). Bargaining power in IJVs is derived from controlling key resources, creating dependency, and can shift when one partner acquires sufficient knowledge or skills to eliminate its dependence on the other (Batra and Dhir, 2024; Inkpen and Beamish, 1997). Both trust and power are expected to mediate the relationship between conflict and IJV performance and stability.

Performance refers to the financial as well as non-financial outcomes of the IJV, including profitability, market share, and innovation (Difalla et al., 2025; Poudel, 2024). It will be assessed using established performance indicators, such as sales growth, profitability, and new product development (Ameyaw et al., 2023; Wang D. et al., 2020). To capture both financial and non-financial aspects, performance in this study is measured using a perceptual, subjective approach, which aligns with the diverse goals of IJV parents (Fey and Beamish, 2001; Larimo et al., 2016). In this study, we rely on this definition performance is defined as the foreign parent’s perceived satisfaction with the IJV’s overall performance, influenced by their control over key resources and activities (Gundelach and Research, 2023; Mjoen and Tallman, 1997; Tetteh et al., 2022a). Shifting to stability, (Inkpen and Beamish, 1997) define instability as the extent to which unplanned, premature major changes occur in a partner relationship. These changes are typically evaluated from the perspective of at least one partner.

This research seeks to fill the identified gap by integrating two foundational theoretical frameworks: (i) Institutional Theory and (ii) Agency Theory. Institutional Theory suggests that organizations operate by both formal and informal regulations and customs that shape behavior, expectations, and legitimacy (Khassawneh and Elrehail, 2022; Lee et al., 2024; Meyer and Rowan, 2021). As partners commence from distinct institutional backgrounds in the perspective of IJVs, thus creating differences in governance practices, strategic priorities, and legal assumptions (Choi et al., 2025; Tetteh et al., 2022b). Later on, these differences increase the probability of conflict and alter how conflict is perceived. Agency Theory defends how joint shared ownership produces uneven access to information, challenges in control, and misaligned incentives (Al-Faryan, 2024; Jensen et al., 1976; Zwalf, 2022).

In IJVs, neither partner possesses absolute control, making power negotiation and oversight at the heart of governance concerns (Reuer et al., 2024), and highlights and often increases these agency-related challenges and tensions (Duan et al., 2025; Gehrisch et al., 2025). By integrating these theoretical viewpoints, we argue that intersection of both institutional divergence and agency misalignment is the place where conflict arises. However, the consequences of such conflict are shaped by governance mechanisms—specifically, through adjustments in trust and power recalibration (Garg and Bingham, 2025; Kesseba, 2026).

Even though enough research has been made to progress this research direction, the role of conflict remains inconclusive in shaping venture outcomes, both in theoretical fragmentation and empirical evidence (Choi et al., 2025; Do et al., 2024; Jin et al., 2025). Herein, we focused rationally on IJVs operating in China – as, China, with its largest economy of today’s world and one of the most active environments for IJVs satisfying the nature of distinctive institutional setting, where formal regulatory (governance) structures exist together with strong relationship-based business practices; which make it relevant for examining how conflict is managed through trust and power; hence, participants here are capable enough with direct and practical experience in managing these partnerships. Particularly, China’s relationship oriented business culture, characterized by guanxi (interpersonal networks), Confucian values, and a collectivist orientation, makes conflict management in IJVs specially dependent on relational mechanisms like trust rather than purely contractual ones. To address this gap, this research aims to explore the following two key research questions:

RQ1: Does conflict affect IJV performance and stability directly, or indirectly through governance mechanisms such as trust and power?

RQ2: How does conflict potential differ from realised conflict in shaping these governance pathways?

By addressing these questions, this study contributes to the literature in several ways. First, instead of treating conflict simply as an input that affects performance, it shows that conflict works more as a trigger that activates governance mechanisms, particularly trust and power. Second, the study considers trust and power together, showing that both relational and structural mechanisms are important in shaping IJV outcomes. Third, it distinguishes between conflict and conflict potential, showing that openness to disagreement behaves differently from actual conflict and mainly works through trust. Finally, by combining Institutional Theory and Agency Theory, the study explains both how conflict arises and how it is managed, providing a clearer understanding of when conflict leads to positive outcomes within IJVs operating in China.

## 2 Theoretical framework and hypothesis development

### 2.1 Conflict and conflict potential in IJVs

This study distinguishes between conflict and conflict potential. Conflict reflects realized disagreements and tensions. Conflict potential, drawing on (Daroczi, 2003), captures openness to divergent views and a willingness to engage disagreement constructively. These items reflect a positive orientation toward engaging disagreement, consistent with prior research on constructive conflict. This interpretation is consistent with constructive controversy and task-conflict research, which shows that opposing views can support learning and problem solving, although the benefit will be conditional on cooperative norms trust and openness (De Dreu and Weingart, 2003; Johnson, 2015).

Accordingly, while conflict reflects actual disagreements between partners, conflict potential refers more to how partners at the individual level approach and engage with those disagreements in a constructive way, rather than being driven by structural factors like institutional differences or goal misalignment. It is related to constructs such as constructive conflict and cognitive diversity but specifically captures the willingness of partners to engage with differing views within IJV relationships (De Dreu and Weingart, 2003; Johnson, 2015). While it’s related to cognitive diversity and constructive controversy, conflict potential is not the same. Cognitive diversity shows differences in knowledge, expertise, or perspectives among partners. Constructive controversy describes a structured process for managing intellectual opposition. Conflict potential, in contrast, captures how willing partners are to engage disagreement constructively in IJV settings. Importantly, while prior IJV research often conceptualises conflict potential in structural terms (e.g., institutional distance or goal misalignment), the present study focuses on a relational, attitudinal perspective based on how partners engage disagreement.

**Figure 1:**
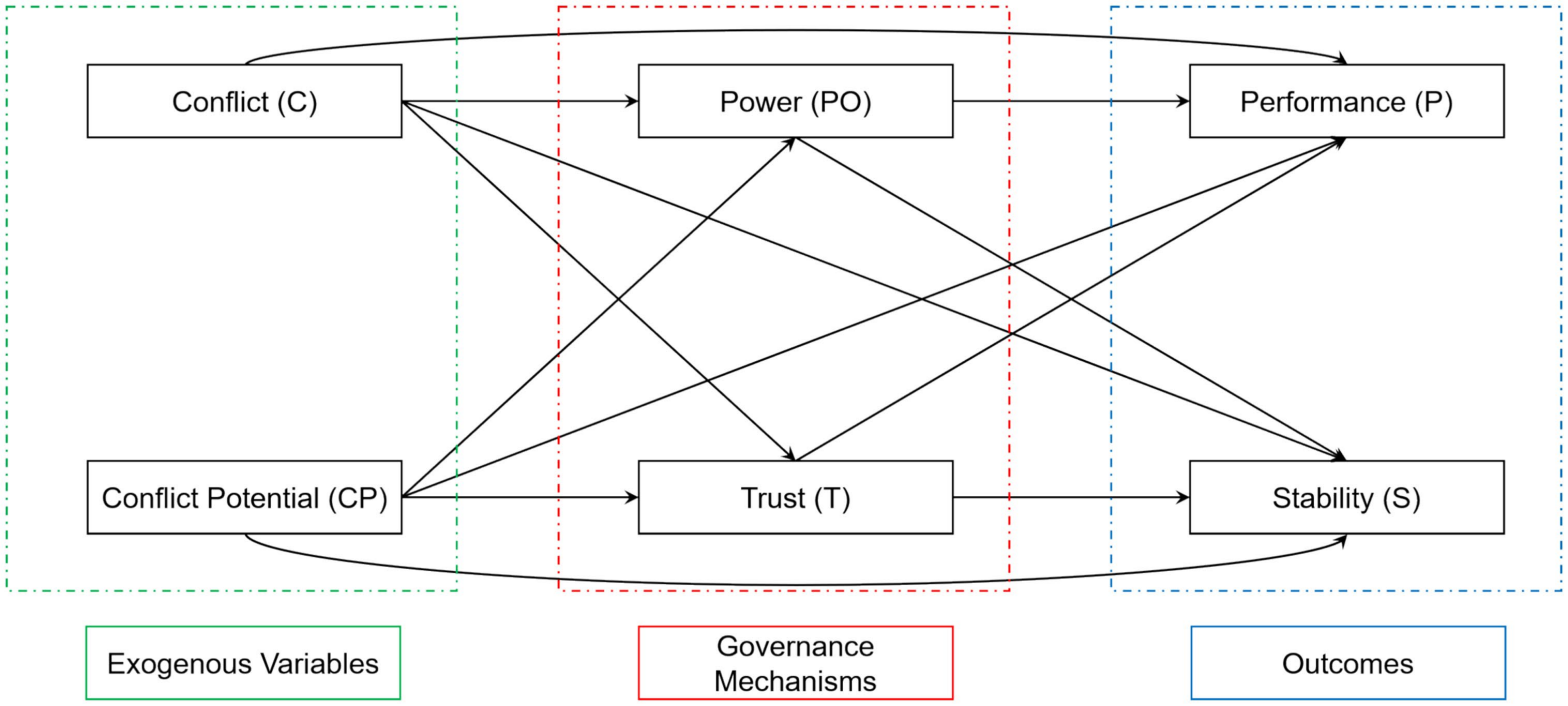
Theoretical framework of this study.

Here in this study, the theoretical framework is presented in Figure 1. The proposed framework conceptualizes conflict (C) and conflict potential (CP) as independent variables, trust (T) and power (PO) as mediating governance mechanisms, and performance (P) and stability (S) as the dependent outcomes of international joint ventures.

A complete summary of the 20 hypotheses and their paths is presented in Table 1.

**Table 1:** Hypothesis testing with SmartPLS.

Hypothesis	Path	SB	STDEV	T value	*p* value	Decision
Conflict effects						
H1	C → P	0.093	0.078	1.194	0.232	Not supported
H2	C → PO	0.230	0.068	3.366	0.001	Supported
H3	C → S	0.153	0.065	2.344	0.019	Supported
H4	C → T	0.363	0.066	5.486	0.000	Supported
Conflict potential effects						
H5	CP → P	−0.001	0.100	0.008	0.994	Not supported
H6	CP → PO	0.153	0.078	1.948	0.051	Not supported
H7	CP → S	0.000	0.062	0.007	0.995	Not supported
H8	CP → T	0.483	0.059	8.126	0.000	Supported
Governance mechanisms						
H9	PO → P	0.153	0.072	2.126	0.034	Supported
H10	PO → S	0.465	0.071	6.559	0.000	Supported
H11	T → P	0.328	0.109	3.020	0.003	Supported
H12	T → S	0.188	0.079	2.359	0.018	Supported
Mediation effects						
H13	C → PO → S	0.107	0.036	2.932	0.003	Supported
H14	CP → PO → S	0.071	0.038	1.861	0.063	Not supported
H15	C → T → P	0.119	0.041	2.902	0.004	Supported
H16	CP → T → P	0.158	0.053	2.969	0.003	Supported
H17	C → PO → P	0.035	0.021	1.706	0.088	Not supported
H18	C → T → S	0.068	0.033	2.035	0.042	Supported
H19	CP → PO → P	0.023	0.017	1.371	0.170	Not supported
H20	CP → T → S	0.091	0.041	2.216	0.027	Supported

Performance in IJVs encompasses financial and non-financial outcomes, including profitability, market share, and innovation. Consistent with prior research (Geringer and Hebert, 1991; Mjoen and Tallman, 1997) performance is measured perceptually to capture diverse strategic objectives of parent firms.

Power reflects control over critical resources and decision-making authority. Agency theory emphasizes that contractual rights and ownership shares allocate control, while bargaining power evolves as dependency structures shift (Inkpen and Beamish, 1997). Conflict may stimulate the renegotiation of authority and resource distribution (Cherbib et al., 2023), leading to clearer governance arrangements.

Stability reflects the degree to which an IJV undergoes strategic reorientation, contract renegotiation, or ownership restructuring. Inkpen and Beamish (1997) conceptualize instability as unplanned structural change, whereas Yan and Zeng (1999) propose a dynamic view recognizing that structural change may reflect adaptation rather than failure. Following this dynamic approach, stability in this study reflects controlled and mutually agreed governance evolution rather than premature disruption.

Trust is the belief that a partner will act reliably, competently, and with goodwill (Boersma et al., 2003). Trust reduces concerns about opportunistic behavior and lowers the need for costly monitoring (Wang L. et al., 2020). In IJVs, where formal contracts cannot anticipate all contingencies, trust functions as relational governance.

Institutional Theory explains that differences in norms and practices between partners lead to conflict. Agency Theory suggests that shared ownership creates misaligned interests and information gaps. Therefore, conflict is expected to influence trust and power, which then affect performance and stability. Conflict may also trigger governance adjustments that stabilize partnerships. Given evidence that constructive conflict may stimulate innovation and learning (Ali et al., 2021), we tested the direct effects of conflict on outcomes:

*H1*: Conflict positively affects IJV performance.

*H2*: Conflict positively affects power recalibration in IJVs.

*H3*: Conflict positively affects IJV stability.

*H4*: Conflict positively affects trust in IJVs.

From an Institutional Theory view, openness to different perspectives (conflict potential) shapes how partners deal with disagreement. From an Agency Theory view, it also affects trust and power between partners. Hence, we further examine the role of conflict potential in shaping relational trust and power governance structures, influencing performance and stability (Boersma et al., 2003). Given that conflict potential may trigger renegotiation and governance clarification, we also tested the effect of conflict potential directly.

*H5*: Conflict potential positively affects IJV performance.

*H6*: Conflict potential positively affects power dynamics in IJVs.

*H7*: Conflict potential positively affects IJV stability.

*H8*: Conflict potential positively affects trust in IJVs.

### 2.2 Trust and power as governance mechanisms

Empirical evidence indicates that trust enhances performance in cross-border partnerships (Aulakh et al., 1996). Constructive conflict management strengthens communication and mutual understanding (Wong et al., 2018), suggesting that conflict and conflict potential may foster trust and performance when partners engage cooperatively. Accordingly, we tested trust and power expected to influence both performance and stability outcomes.

*H9*: Power positively affects IJV performance.

*H10*: Power positively affects IJV stability.

*H11*: Trust positively affects IJV performance.

*H12*: Trust positively affects IJV stability.

The integrated theoretical framework suggests that conflict and conflict potential influence performance and stability indirectly through trust and power dynamics (Boersma et al., 2003). Institutional divergence increases the likelihood of disagreement, while agency dynamics shape how conflict is managed. Trust reduces opportunism and enhances coordination; power recalibration clarifies authority and stabilizes governance.

### 2.3 Mediating mechanisms

Following Agency Theory, trust and power are expected to mediate these relationships by reducing opportunism and clarifying control. After our extensive literature review, we found that conflict and conflict potential not only influence IJV outcomes directly, but also influence them indirectly based on these mediating relations, as hypothesized in this section. We can hypothesize how conflict and conflict potential are translated into performance and structural outcomes while mediating through power and trust and vice versa. The research of Inkpen and Beamish (1997) maintained that bargaining power progresses as partners adjust dependence and control over time, in the same context, Cherbib et al. (2023) maintained that conflict might trigger governance recalibration that stabilizes partnerships.

*H13*: Power mediates relationship between conflict and IJV stability.

Since governance structures adapt to manage uncertainty and institutional differences in IJVs, as Choi et al. (2025) studied the role of institutional environment on contract design has been significant as governance mechanisms of IJVs. Hence, conflict potential may influence stability through power. So,

*H14*: Power mediates relationship between conflict potential and IJV stability.

Trust, defined as confidence in a partner’s trustworthiness and goodwill (Boersma et al., 2003), reduces unscrupulousness and coordination costs, thereby improving performance (Aulakh et al., 1996). So, Trust mediates relationship between conflict and performance because constructive conflict improves understanding of each other as well as communication. Similarly, conflict potential may also enhance performance indirectly through trust.

*H15*: Trust mediates relationship between conflict and IJV performance.

*H16*: Trust mediates relationship between conflict potential and IJV performance.

Since it is well understood that Conflict can force partners to renegotiate control over resources and decision-making authority, leading to better performance. As we described in the introduction of this research paper that power in IJVs is based on holding critical resources and establishing dependency. This power dynamic can change if a partner gains enough expertise or knowledge to reduce or eliminate its reliance on the other party (Batra and Dhir, 2024; Inkpen and Beamish, 1997). Hence, Power may also potentially mediate the relationship between conflict and IJV performance, while Trust mediates relationship between conflict and stability by reducing fears of opportunistic behavior and strengthening commitment between partners (Aulakh et al., 1996; Boersma et al., 2003).

*H17*: Power mediates relationship between conflict and IJV performance.

*H18*: Trust mediates relationship between conflict and IJV stability.

Inkpen and Beamish (1997) maintained that Power mediates relationship between conflict potential and performance, particularly when constructive disagreement enables partners to adjust authority structures. Finally, Daroczi (2003) believes that trust, as openness to differing perspectives, fosters relations by enhancing the venture’s ability to remain stable. So, trust may mediate the relationship between conflict potential and IJV stability.

*H19*: Power mediates relationship between conflict potential and IJV performance.

*H20*: Trust mediates relationship between conflict potential and IJV stability.

## 3 Methodology

A quantitative methodology was employed in this research to investigate both the direct and mediated connections among the variables under study.

### 3.1 Research instruments

This study investigates how conflict and conflict potential affect performance, stability, and trust in international joint ventures. A 7-point Likert scale (1 = strongly disagree, 7 = strongly agree) was used to assess all constructs. In total, 22 items were used to measure six key constructs, all evaluated using items from previous research, demonstrating strong reliability across the board. The C construct was assessed using six items adapted from Habib (1987) and Dyer and Song (1997) yielding a reliability coefficient of 0.74. The CP construct consisted of five items sourced from Daroczi (2003), with a reliability value of 0.76. For P, three items from Lin and Germain (1998) were used, following guidelines from Geringer and Hebert (1991), producing a reliability score of 0.85. The PO construct was measured using three items from Lin and Germain (1998) with a reliability score of 0.80. The T construct was evaluated with three items from Aulakh et al. (1996) and Boersma et al. (2003) showing a reliability coefficient of 0.77. Finally, the S construct was assessed using 2 items from Yan and Zeng (1999), with a reliability coefficient of 0.85. The use of a two-item construct is acceptable in PLS-SEM when indicators are theoretically grounded and demonstrate strong reliability and loadings.

### 3.2 Questionnaire outcome

After a careful literature review (Carter and Roger, 2008; Shela et al., 2023), we took out validated scales to develop a well-structured questionnaire, and then we got it approved by the internal expert panel of our college. Then we performed a pre-test of individuals involving 46 participants (refer to Table 2), which helped us to remove 1 item that were of less significance with a threshold of 0.7 or less. Items were removed based on standardized indicator (outer) loadings below 0.70 in the pilot measurement model, consistent with commonly used PLS-SEM guideline.

**Table 2:** Outer loadings result on the pre-test.

	Conflict	Conflict potential	Performance	Power	Stability	Trust
C1	0.716					
C2	0.774					
C3	0.874					
C4	0.864					
C5	0.886					
C6	0.610					
C7	0.704					
CP1		0.894				
CP2		0.863				
CP3		0.867				
CP4		0.748				
CP5		0.701				
P1			0.893			
P2			0.914			
P3			0.904			
PO1				0.863		
PO2				0.883		
PO3				0.879		
S1					0.933	
S2					0.933	
T1						0.810
T2						0.909
T3						0.902

### 3.3 Sampling method

The non-probability sampling approach was used to collect data from professionals directly involved in IJVs in China. Due to the absence of a comprehensive sampling frame and the difficulty of accessing IJV managers, this approach was appropriate for reaching knowledgeable respondents (Opuala-Charles et al., 2022). A combination of snowball, quota, and convenience sampling was applied. Snowball sampling facilitated access through professional networks and WeChat business groups. Quota sampling ensured representation across organizational roles (e.g., entrepreneurs, managers, employees) and firm characteristics (e.g., firm size and age) (Futri et al., 2022). Convenience sampling allowed inclusion of accessible respondents who met the study criteria. Herein, focusing on Chinese IJVs provided consistent institutional context, reducing variation related to differences in cross-country and allowing reflection of governance mechanisms (trust and power) within comparable conditions. China’s relationship-based environment, influenced by guanxi and long-term collaborative orientation, makes trust and power dynamics especially important for evaluating the proposed governance mechanisms.

### 3.4 Data administration and response screening

The online questionnaire was distributed to participants from February 24 to April 25, 2024. The survey targeted a diverse group of individuals, including entrepreneurs, employees, and business owners from micro, small, medium, and large enterprises. Data were collected using an online questionnaire, available in Chinese, English, and Arabic, to accommodate different groups across China. The survey was created using Microsoft Forms and distributed mainly through WeChat to reach a broad audience of professionals in China. Following the guidelines of the American Psychological Association and the Institutional Review Board, we guaranteed that informed consent was given by participants, and throughout this study, their confidentiality was well-respected (Qin et al., 2019; Serpico, 2024). We received 208 responses, which we rechecked to ensure that the same participant had not responded twice by checking the standard deviation among the responses, and 9 responses with 0 deviations were excluded. Hence, the 199 valid responses covering 22 questions and 6 sections were brought into consideration in this study.

### 3.5 Data analysis

Normality and distribution of the data were assessed before selecting the appropriate analysis model. Because the dataset did not exhibit a normal distribution, Partial Least Squares Structural Equation Modeling (PLS-SEM) was employed, a method known for handling non-normal data effectively (Hair and Alamer, 2022). This approach was deemed suitable for our sample of 199, with 6 indicator variables and 22 items (Sarstedt and Liu, 2024). The analysis procedure included several key steps: initially, the data distribution was reviewed, followed by checks for the validity of the factors (Zhao et al., 2025). After that, we tested the reliability of the results and assessed both the measurement model and discriminant validity. These analyses were conducted using SPSS 29 and SmartPLS 4 software (Cheah et al., 2024; George and Mallery, 2024).

### 3.6 Common method bias

In order to evaluate the presence of common method bias in this research, we examined both HTMT ratios and VIF values. Based on the guidelines from Nitzl (2016), common method bias is considered a concern when the correlations between the primary constructs exceed a threshold of 0.90. In our analysis, the correlation values were all well below this limit, with the highest correlation observed being 0.688 (refer to Table 3). This suggests that common method bias is not an issue in our data. Additionally, we assessed the inner VIF values as another check for common method bias. As per the guidelines from Kock (2017), VIF value above 3.30 indicates potential common method bias. In this study, the highest recorded VIF value was 2.003 (refer to Table 4), which is significantly below the threshold, further supporting the conclusion that common method bias is not a concern.

**Table 3:** Evaluation of discriminant validity using Fornell–Larcker criterion and HTMT ratios.

	C	CP	P	PO	S	T
Fornell–Larcker criterion						
C	0.781					
CP	0.335	0.818				
P	0.314	0.262	0.904			
PO	0.284	0.228	0.293	0.875		
S	0.379	0.27	0.438	0.572	0.933	
T	0.525	0.603	0.429	0.347	0.429	0.875
Herterotrait–Monotrait criterion						
C						
CP	0.367					
P	0.351	0.273				
PO	0.326	0.261	0.334			
S	0.433	0.31	0.503	0.672		
T	0.594	0.688	0.49	0.407	0.501	

**Table 4:** Collinearity statistics (VIF)—inner model matrix.

	P	PO	S	T
C	1.404	1.126	1.404	1.126
CP	1.572	1.126	1.572	1.126
P				
PO	1.156		1.156	
S				
T	2.003		2.003	

### 3.7 Respondents’ profile

The demographics of all 199 respondents have been summarized in Table 5. Among them, 59.8% of respondents were female, and 40.2% were male. If classified based on age, the majority was between 35 and 54 years old was 43.7%, while the 25–34 years group was only 33.7%. 55.3% of the respondents had a Master’s degree or higher, and 40.7% held a Bachelor’s degree. Regarding position, 28.1% were Junior Managers, 29.1% were Middle Managers, and 18.1% were in Senior Management. As for company age, most respondents worked in companies aged 7–10 years (27.1%) and more than 15 years (25.6%). In terms of company size, 39.2% were from companies with 50–249 employees, and 26.1% were from companies with 250 or more employees.

**Table 5:** Demographic analysis of respondents.

	Frequency	Percent
Gender		
Male	80	40.2%
Female	119	59.8%
Age (years)		
18 to 24	39	19.6%
25 to 34	67	33.7%
35 to 54	87	43.7%
55+	6	3.0%
Education		
Master and above	110	55.3%
Bachelor	81	40.7%
Diploma	7	3.5%
High school and under	1	0.5%
Position		
Staff	49	24.6%
Junior Manager	56	28.1%
Middle Manager	58	29.1%
Senior Management	36	18.1%
Age of company (years)		
15+	51	25.6%
11–15	35	17.6%
7–10	54	27.1%
3–6	32	16.1%
<3	27	13.6%
Number of employees		
<10	11	5.5%
10 to 49	54	27.1%
50 to 249	78	39.2%
250+	52	26.1%

Source: Compilation derived from data processed through SPSS version 29 (Sample size = 199).

## 4 Data analysis and results

### 4.1 Descriptive statistics

Mean, Std. deviation, Skewness, and Kurtosis for each construct are presented in Table 6. PO has the highest mean score, suggesting a stronger overall tendency toward higher ratings. The standard deviation figures show the spread of responses, with T showing the most variation and PO the least, reflecting differences in response consistency. Skewness values are mostly negative, with S exhibiting the most negative skewness (−1.192), indicating a pronounced leftward skew. The kurtosis values are generally positive, with S showing the highest value (2.739), indicating a more peaked distribution with heavier tails, while the other constructs show less extreme kurtosis, indicating distributions closer to normal.

**Table 6:** Mean, std. deviation, Skewness, and Kurtosis values for each construct.

	N	Mean	Std. deviation	Skewness	Kurtosis
C	199	5.199	1.149	−1.054	1.039
CP	199	5.192	1.166	−0.599	0.114
T	199	5.280	1.220	−0.842	0.566
PO	199	5.938	0.836	−0.760	0.722
P	199	5.567	1.091	−0.887	0.587
S	199	5.625	0.999	−1.192	2.739

Table 7 presents the Kolmogorov–Smirnov and Shapiro–Wilk test results (Konopatov et al., 2024; Steinskog et al., 2007), both indicating significant departures from normality (*p* < 0.05). These results highlight that parametric approaches are unsuitable because the normality assumptions are not met. To overcome this challenge, the study utilizes PLS-SEM, a method that is resistant to non-normal data distributions and works well with smaller sample sizes. In contrast to traditional covariance-driven SEM, PLS-SEM focuses on boosting the proportion of variance accounted for, is well-suited for intricate frameworks containing both formative and reflective constructs, and relies on bootstrapping to test the significance of path coefficients. These features make PLS-SEM an effective technique for managing non-normal data while maintaining the validity of the analysis (Hair et al., 2012).

**Table 7:** Normality tests: Kolmogorov-Smirnova and Shapiro–Wilk.

Items	Kolmogorov-Smirnova			Shapiro–Wilk		
	Statistic	df	Sig.	Statistic	df	Sig.
C1	0.215	199	<0.001	0.906	199	<0.001
C2	0.227	199	<0.001	0.875	199	<0.001
C3	0.245	199	<0.001	0.871	199	<0.001
C4	0.257	199	<0.001	0.84	199	<0.001
C5	0.228	199	<0.001	0.86	199	<0.001
C6	0.16	199	<0.001	0.915	199	<0.001
CP1	0.186	199	<0.001	0.916	199	<0.001
CP2	0.202	199	<0.001	0.909	199	<0.001
CP3	0.191	199	<0.001	0.915	199	<0.001
CP4	0.245	199	<0.001	0.876	199	<0.001
CP5	0.202	199	<0.001	0.886	199	<0.001
T1	0.234	199	<0.001	0.867	199	<0.001
T2	0.224	199	<0.001	0.872	199	<0.001
T3	0.219	199	<0.001	0.895	199	<0.001
PO1	0.233	199	<0.001	0.832	199	<0.001
PO2	0.233	199	<0.001	0.837	199	<0.001
PO3	0.248	199	<0.001	0.856	199	<0.001
P1	0.228	199	<0.001	0.881	199	<0.001
P2	0.224	199	<0.001	0.887	199	<0.001
P3	0.251	199	<0.001	0.872	199	<0.001
S1	0.253	199	<0.001	0.866	199	<0.001
S2	0.265	199	<0.001	0.857	199	<0.001

Source: Compiled by the authors from data analyzed using SPSS version 29.

**Figure 2:**
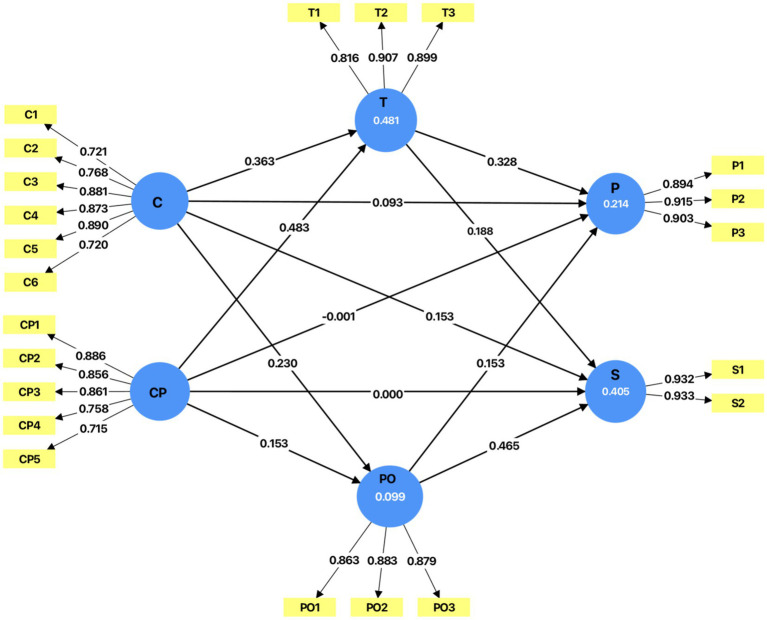
Evaluation of inner and outer models using SmartPLS 4.1.0.7.

The inner and outer models, presented in Figure 2 show the outer loadings of items and the associations among variables in the PLS-SEM evaluation, with findings showing robust affirmative relationships between P and S, P and T, and P and PO. Similarly, T shows positive relationships with both P and S, while PO exhibits positive connections with P and Sas well. A negative relationship is observed between CP and P, and a highly significant relationship is found between CP and S. Hence, it can be undertaken that P plays a central role in driving positive outcomes across several constructs, while the negative effect of CP on P warrants deeper investigation.

### 4.2 Measurement model assessment

As shown in Table 8, all items across the displays statistically significant outer weights, having *p*-values below 0.01 (***), confirming their substantial contribution to the respective constructs. The variance inflation factor (VIF) values remain well under 5, suggesting that the model has no issues related to collinearity or multicollinearity (Aguirre-Urreta et al., 2016; Sarstedt et al., 2020; Shela et al., 2023).

**Table 8:** Model assessment. SmartPLS.

Variables	Items	Outer weights	*p*-value	VIF
Conflict (C)				
The partners often argue over operational issues	C1	0.185	0.000	1.644
The operational disagreements between the partners are intense.	C2	0.194	0.000	1.853
There is little or no conflict between the partners.	C3	0.232	0.000	3.157
Representatives from both firms assess the importance of decisions similarly.	C4	0.185	0.000	3.439
Representatives from both firms share the same values.	C5	0.223	0.000	3.533
Representatives from both firms differ on the joint venture’s goals.	C6	0.213	0.000	1.543
Conflict potential (CP)				
I prefer working with a foreign partner with different business views.	CP1	0.276	0.000	3.8
Different manager perceptions can solve business problems.	CP2	0.248	0.000	3.463
Diverse perspectives aid the joint venture in adapting to change and overcoming challenges.	CP3	0.298	0.000	2.289
Conflicts between managers may build trust over time.	CP4	0.215	0.000	2.164
Some conflict between managers is better than none.	CP5	0.174	0.000	2.086
Performance (P)				
We are satisfied with the personal partnership we have with our external collaborator.	P1	0.397	0.000	2.225
We are satisfied with the overall partnership we have with our external collaborator.	P2	0.367	0.000	2.946
The performance of the joint venture is financially satisfactory	P3	0.343	0.000	2.819
Power (PO)				
We have more power than our foreign partner.	PO1	0.401	0.000	1.847
We influence decisions more than our foreign partner.	PO2	0.358	0.000	2.286
We are dependent on our foreign partner	PO3	0.384	0.000	2.153
Stability (S)				
Joint venture regularly updates its strategic goals.	S1	0.535	0.000	2.209
Joint venture often alters its ownership structure or leadership.	S2	0.537	0.000	2.209
Trust (T)				
High trust with our foreign partner.	T1	0.326	0.000	1.727
Both firms trust each other to follow the joint venture contract.	T2	0.398	0.000	2.506
We and our partner firm typically do not trust the information shared between us.	T3	0.415	0.000	2.333

Table 9 presents the outer loadings and reliability measures for each construct. It summarises reliability as well as validity analysis conducted with SmartPLS 4.1.0.7, featuring outer loadings, Cronbach’s alpha (*α*), Dijkstra-Henseler’s rho (rho_A), composite reliability (rho_C), and average variance extracted (AVE); each outer loading is above 0.70 (refer to Table 9), confirming strong item-construct relationships (Cheah et al., 2021). As can be seen, Cronbach’s alpha, rho_A, and rho_C are above 0.70, showing consistency in the results. Each construct’s AVE surpasses 0.50, supporting convergent validity. In the case of the stability construct, the two indicators show high and consistent outer loadings, resulting in a high AVE, which reflects strong internal consistency rather than a measurement artefact. Collectively, the results confirm the measurement model is stable and appropriate for proceeding with structural analysis.

**Table 9:** Average variance extracted (AVE), composite reliability, and Cronbach’s alpha using SmartPLS.

Construct	Items	Outer loading	alpha	rho_a	rho_c	AVE
Conflict	C1	0.721	0.895	0.9	0.92	0.66
	C2	0.768				
	C3	0.881				
	C4	0.873				
	C5	0.89				
	C6	0.72				
Conflict potential	CP1	0.886	0.876	0.898	0.909	0.669
	CP2	0.856				
	CP3	0.861				
	CP4	0.758				
	CP5	0.715				
Performance	P1	0.894	0.888	0.892	0.93	0.817
	P2	0.915				
	P3	0.903				
Power	PO1	0.863	0.848	0.849	0.908	0.766
	PO2	0.883				
	PO3	0.879				
Stability	S1	0.932	0.85	0.85	0.93	0.87
	S2	0.933				
Trust	T1	0.816	0.847	0.861	0.907	0.766
	T2	0.907				
	T3	0.899				

rho_a and rho_c indicate composite reliability, AVE refers to average variance extracted, and Alpha represents Cronbach’s alpha.

Discriminant validity was assessed using two methods, as it was presented in Table 3: the Fornell–Larcker and HTMT criteria. The Fornell–Larcker criterion verifies unique validity whenever the square root of each construct’s AVE (bold values on the diagonal) exceeds the associations with alternative factors (values not on the diagonal), a condition satisfied by every factor. The HTMT criterion, a stricter test, requires construct ratios to be below 0.85; all values comply (Shela et al., 2023). Both methods establish that the constructs are empirically distinct. Importantly, the HTMT results confirm that conflict and conflict potential are empirically distinct constructs.

### 4.3 Structural model analysis

**Figure 3:**
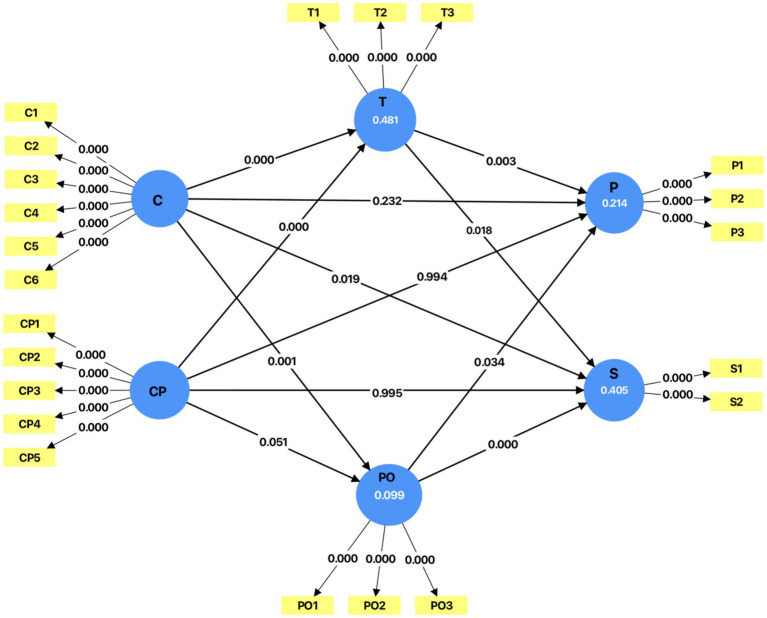
PLS-SEM Bootstrapping analysis by SmartPLS.

The bootstrapping results from SmartPLS are presented in Figure 3 and Table 1, with the evaluation of hypotheses using standardized coefficients, standard errors, t-statistics, and *p*-values; Standardized beta coefficients indicate both the strength and direction of the relationships: positive values suggest a direct link, while negative coefficients reflect an inverse association. Hypotheses with p-values less than 0.05 are regarded as statistically significant.

The analysis revealed several key findings. C showed a positive but non-significant impact on P (Original sample = 0.093, T = 1.194, *p* = 0.232), leading to the rejection of H1. However, C did have a significant positive impact on PO (Original sample = 0.23, T = 3.366, *p* = 0.001), confirming H2, and also showed a positive impact on S (Original sample = 0.153, T = 2.344, *p* = 0.019), supporting H3. C had a strong positive effect on T (Original sample = 0.363, T = 5.486, *p* < 0.001), therefore confirming H4. CP did not significantly influence P (Original sample = −0.001, T = 0.008, *p* = 0.994), leading to rejection of H5, but it did have a marginally significant effect on PO (Original sample = 0.153, T = 1.948, *p* = 0.051), partially confirming H6. CP had no significant effect on S (Original sample = 0.0, T = 0.007, *p* = 0.995), which led to the rejection of H7, but showed a significant positive impact on T (Original sample = 0.483, T = 8.126, *p* < 0.001), confirming H8. PO positively influenced P (Original sample = 0.153, T = 2.126, *p* = 0.034), confirming H9, and also demonstrated a strong positive effect on S (Original sample = 0.465, T = 6.559, *p* < 0.001), confirming H10. T had a significant positive effect on P (Original sample = 0.328, T = 3.02, *p* = 0.003), supporting H11, and also influenced S (Original sample = 0.188, T = 2.359, *p* = 0.018), confirming H12.

### 4.4 Mediation analysis

The mediation analysis indicated significant indirect effects, including C → PO → S (Original sample = 0.107, T = 2.932, *p* = 0.003), confirming H13, and a marginally significant effect of CP → PO → S (Original sample = 0.071, T = 1.861, *p* = 0.063), providing partial support for H14. C → T → P showed a significant indirect effect (Original sample = 0.119, T = 2.902, *p* = 0.004), confirming H15, as did CP → T → P (Original sample = 0.158, T = 2.969, *p* = 0.003), confirming H16. The indirect effect of C → PO → P was marginally significant (Original sample = 0.035, T = 1.706, *p* = 0.088), partially supporting H17, and C → T → S showed a significant indirect effect (Original sample = 0.068, T = 2.035, *p* = 0.042), confirming H18. CP → PO → P was not significant (Original sample = 0.023, T = 1.371, *p* = 0.170), leading to the rejection of H19, while CP → T → S showed a significant indirect effect (Original sample = 0.091, T = 2.216, *p* = 0.027), confirming H20 (Henseler et al., 2009; Shela et al., 2023; Wetzels et al., 2009).

### 4.5 Goodness of fit

The results shown in Table 10 indicate the model’s fit in the context of PLS-SEM. The *R*^2^ values indicate different levels of explanatory strength for the endogenous latent variables. Specifically, the *R*^2^ for P is 0.214, for PO is 0.099, for S is 0.405, and for T is 0.481. While some of these R^2^ values are relatively lower (e.g., PO), they still reflect a reasonable level of explained variance, especially for S and T. The Q^2^ values are used to evaluate the predictive relevance of the model, which are Q^2^ for P is 0.093, for PO is 0.077, for S is 0.147, and for T is 0.459. Since these values are all greater than zero, they suggest that the model has predictive power across the different latent variables, with T showing the strongest predictive relevance (Henseler et al., 2009). In addition, the standardized root mean square residual (SRMR) is 0.069, which falls below the widely recognized cutoff of 0.08. These results agree with what has been modelled in the systematic review of (Shela et al., 2023). The model proposed in this study offers an acceptable goodness of fit, some of those aspects even proved stronger correlation than others, in particular for Trust, in alignment with previous literature (Wetzels et al., 2009).

**Table 10:** Robust model fit. SmartPLS.

	P	PO	S	T
R2	0.214	0.099	0.405	0.481
Q^2^predict	0.093	0.077	0.147	0.459
SRMR	0.069			

## 5 Discussion

Herein, we investigated in what manner conflict and conflict potential affect international joint venture (IJV) performance and stability via the governance mechanisms of trust and power. By incorporating Institutional Theory and Agency Theory, we conceptualized conflict as a structural consequence of institutional differences within the Chinese IJV context and shared ownership, and examined whether its consequences are direct or conditional upon governance responses.

The outcomes indicate that conflict does not directly lead to better performance, as the direct path from conflict to performance was not established. This finding is generally in line with prior researches that highlights the role of trust in imprving coordination and reducing opportunistic behavior in IJVs, suggesings that conflict itself is not automatically beneficial (Aulakh et al., 1996; Boersma et al., 2003); However, this theory is different than those argueing otherwise, which believe that constructive conflict can directly enhance performance by nurturing learning and innovation (Ali et al., 2021; Wong et al., 2018), whereas we found that such benefits are not immediate but are depending on governance responses.

This challenges simplified assumptions that disagreement is inherently valuable because it fosters innovation or learning. Instead, the significance of conflict lies in its capability to trigger changes in how the partnership is managed—particularly by improving trust and power recalibration—and influencing performance and stability indirectly through these mechanisms. Thus, conflict yields positive results only when it activates relational and structural governance responses.

Likewise, Conflict potential also showed a similar pattern. Even though it did not directly enhance performance or stability, it was a strong indicator of trust and indirectly affected outcomes through relational pathways. This indicates that openness to contrasting opinions and viewpoints is critical, suggesting that how partners approach disagreement matters more than disagreement itself.

Trust emerges as a central relational mechanism, significantly improving both performance and stability while mediating key relationships. This reinforces earlier findings that trust plays a key role in reducing uncertainty and opportunistic behavior in IJVs, particularly when formal contracts cannot fully specify all contingencies (Aulakh et al., 1996; Boersma et al., 2003).

Consistent with Agency Theory (Al-Faryan, 2024; Jensen et al., 1976), trust reduces perceived opportunism and coordination costs, enabling partners to transform institutional differences into collaborative advantage. Similarly, power recalibration performed a critical structural role in maintaining stability within the joint venture, which shares some consistency with prior work which suggested bargaining power and control are central to IJV evolution and stability (Inkpen and Beamish, 1997). Conflict appears to prompt renegotiation of authority and control over resources, thereby clarifying governance structures and strengthening organizational resilience. At the same time, the IJVs in Chinese context likely influences these findings as the business relationships tend to rely more heavily on relational norms and long-term orientation in China, which may strengthen the role of trust observed in this study. These results suggests that the strong effect of trust may be more pronounced in similar relational environments. This helpout explaining why trust shows a particularly strong effect on both performance and stability in our model. Simultaneously, the indirect role of conflict through trust and power may be more pronounced in those such relationship-oriented environments compared to more contract-driven settings.

Generally, these results suggest that the relationship between conflict and IJV outcomes is better understood as conditional rather than direct. While earlier researches often treated conflict as either beneficial or harmful – our findings indicate that its impact depends on how it is interpreted into governance mechanisms. Taken together, these insights support a reinterpretation of conflict within IJV governance. Rather than viewing conflict as inherently destructive, it may catalyze governance practices. Institutional gaps and agency-related tensions may generate disagreement, but joint ventures that address these challenges through trust-building and power realignment are more likely to achieve stability and satisfactory performance. In this sense, conflict operates as a governance trigger rather than a direct performance driver. Hence, while the general patterns may be relevant to other IJV contexts, the relative importance of trust and power may differ in environments where governance is more contract-based rather than relationship-driven.

This research contributes to international business theory in three ways. First, it resolves disagreements in perspectives in the IJV literature by showing that the effects of conflict depend on specific conditions and are mediated. Second, it combines institutional and agency explanations into a comprehensive model linking external influences and internal governance processes. Third, it separates conflict from conflict potential, indicating that the way partners approach openness in relationships controls how disagreements are managed.

From a managerial perspective, the findings provide several implications.

- Conflict does not directly enhance performance but operates through governance mechanisms; hence, managers should not seek to eliminate conflict within IJVs, but should formalize mechanisms for structured disagreements. It may include formal escalation protocols and/or joint decision-making forums, mainly to channel disagreements constructively.
- Given the positive effect of trust on both performance and stability, managers should prioritize trust-building, and periodically recalibrate and regularly review decision rights and leadership roles to reduce opportunism and enhance coordination.
- As power dynamics strongly influence stability, managers should periodically reassess and rebalance control over key resources and decision rights through governance reviews and contractual renegotiation mechanisms.

This study is subject to several limitations. The cross-sectional design restricts causal inference and does not capture the dynamic evolution of trust and power over time. Additionally, the use of perceptual, single-respondent data may introduce common method bias regardless of statistical controls. Moreover, conflict potential is measured as a relational–attitudinal construct rather than as structural factors such as institutional distance, which future research may incorporate. Furthermore, the stability construct was measured using only two items, which may limit the depth of measurement and could be expanded in future research. Future research should employ longitudinal and multi-source designs to examine how governance mechanisms evolve across different institutional contexts. Further investigation into moderating factors such as institutional distance, ownership asymmetry, and cultural differences would also enhance understanding of when conflict strengthens or destabilizes IJVs.

## 6 Conclusion

This research advances the comprehension of IJVs by showing that conflict is neither inherently harmful nor automatically beneficial; instead, its value depends on how it is managed through governance mechanisms. Integrating Institutional Theory and Agency Theory, we found that institutional divergence and agency differences misalignment lead to disagreement, yet ultimate effects on performance and stability outcomes are largely shaped by whether conflict triggers trust and power recalibration. The findings suggest that conflict does not directly enhance performance; rather, its benefits are realized indirectly through relational trust and clearer governance frameworks. This study further highlights how managers’ openness to disagreement is crucial by clearly distinguishing between conflict and conflict potential. These results recast conflict as a catalyst for governance change, which, when managed with effective relational and structural mechanisms, can foster adaptive stability and ongoing success in IJVs. However, it should be noted that the exclusive focus on Chinese IJVs might introduces potential cultural and institutional bias; Specifically, these findings reflect governance patterns shaped by guanxi networks, long-term orientation, and collectivist values, which do amplify the role of trust in mitigating conflict. In contrast, IJVs in contract-driven enviroments (e.g., Western economies) could rely more heavily on formal contractual mechanisms, where power recalibration could play a stronger role than trust comparatively. Therefore, the suggested model should be interpreted within its cultural and institutional context, and hence these findings of this research paper may reflect governance patterns that are specifically to relationship-oriented business environments (as in China) and might not fully be apply to countries with different regions with differences. Future rsearch can test this model across multiple countries to assess the generalizability of the results.

## Data Availability

The raw data supporting the conclusions of this article will be made available by the authors, without undue reservation.
